# Linkage mapping and QTL analysis of growth traits in *Rhopilema esculentum*

**DOI:** 10.1038/s41598-021-04431-0

**Published:** 2022-01-10

**Authors:** Bailing Chen, Yulong Li, Meilin Tian, Hao Su, Wei Sun, Yunfeng Li

**Affiliations:** Liaoning Ocean and Fisheries Science Research Institute, 50 Heishijiao St., Dalian, 116023 Liaoning China

**Keywords:** Animal breeding, Genetic association study, Genome, Sequencing

## Abstract

*R. esculentum* is a popular seafood in Asian countries and an economic marine fishery resource in China. However, the genetic linkage map and growth-related molecular markers are still lacking, hindering marker assisted selection (MAS) for genetic improvement of *R. esculentum*. Therefore, we firstly used 2b-restriction site-associated DNA (2b-RAD) method to sequence 152 *R. esculentum* specimens and obtained 9100 single nucleotide polymorphism (SNP) markers. A 1456.34 cM linkage map was constructed using 2508 SNP markers with an average interval of 0.58 cM. Then, six quantitative trait loci (QTLs) for umbrella diameter and body weight were detected by QTL analysis based on the new linkage map. The six QTLs are located on four linkage groups (LGs), LG4, LG13, LG14 and LG15, explaining 9.4% to 13.4% of the phenotypic variation. Finally, 27 candidate genes in QTLs regions of LG 14 and 15 were found associated with growth and one gene named *RE13670* (sushi, von Willebrand factor type A, EGF and pentraxin domain-containing protein 1-like) may play an important role in controlling the growth of *R. esculentum*. This study provides valuable information for investigating the growth mechanism and MAS breeding in *R. esculentum.*

## Introduction

Edible jellyfish *R. esculentum* distributes in the northwest Pacific Ocean and is a popular seafood in Asian countries, especially in China^1,2^. *R. esculentum* is rich in protein and minerals while low in calories and fats, making it an ideal nutritive ingredient for developing oral formulations, functional food and nutricosmetics^3^. In addition, the collagen peptides from *R. esculentum* can accelerate the wound healing process of mice^4^ and have antihypertensive activity^5^, suggesting it can be applied in pharmaceutical industry. The edibility, nutritional value and medicinal properties of *R. esculentum* make it an economical fish resource and widely farmed in aquaculture systems of China^2^. However, overfishing decreased the population number of *R. esculentum* and caused the scarcity of natural resource^6^. To sustainable development and utilization of *R. esculentum* resources, it’s necessary to investigate the genetic background and genetic improvement.

Genetic linkage maps play an important role in studies of genome and genetic^7^. The advance of the 2b-RAD method attracts many researchers’ attention and has accelerated the identification of SNP markers for constructing genetic linkage maps in farmed fishes, such as *Carassius auratus*^8^, *Cyprinus carpio haematopterus*^9^, *Hypophthalmichthys nobilis*^10^ and *Hemibagrus wyckioides*^11^. The average marker interval of these linkage maps was between 0.44 and 0.87 cM and helped mapping QTLs of interested growth traits for genetic breeding^8–11^. Based on the constructed linkage maps, many QTLs related to economic traits, such as body weight, body length, nutritional metabolisms and sex have been identified^7,10–12^. For example, one QTL related to body weight in *Apostichopus japonicas*, explaining 11.8% of the phenotypic variation^7^.

Although the *R. esculentum* genome has been released^1,13^, the genetic linkage map and QTL for growth in *R. esculentum* have not been reported yet. In previous studies, the researchers only identified some markers in *R. esculentum*, such as microsatellite for detection of genetic diversity and conservation of germplasm resources^14^ and SNPs and simple sequence repeats (SSRs) for assisting MAS breeding^15^. To improve the growth of *R. esculentum* through MAS breeding, we identified SNP markers by 2b-RAD method and constructed the first genetic linkage map of *R. esculentum*. Furthermore, we detected some QTLs and genes related to growth traits of *R. esculentum* based on the linkage map. This study will provide some insights for investigating the growth mechanism and genetic research of *R. esculentum* in the future.

## Results

### 2b-RAD sequencing

A total of 1539 million raw reads were generated by 2b-RAD sequencing, including 19 million from the male and female parents and 1520 million from the offspring (Table 1). The ratio of clean reads to raw reads is higher than 93.63% and the alignment rate of the clean reads to the genome reference sequence of *R. esculentum* was close to 63% (Table 1). The tag number of female and male parents was both higher than that of F1 offspring while the tag depth was both lower than that of F1 offspring.

**Table 1:** Summary of 2b-RAD sequencing.

	Raw reads	Clean reads	Alignment rate	Tag number	Tag depth
Male parent	9,594,032	8,982,914	62.95%	36,765	154
Female parent	9,594,032	9,109,329	62.17%	36,503	155
F1 offspring (mean)	10,556,728	10,056,703	63.13%	35,624	178

### SNP markers and linkage mapping

A total of 9100 SNP markers were generated with the transition to transversion (TS/TV) ratio of 1.48. After filtering, 6674 SNP markers can be used for constructing the linkage map. Through the neighbor-joining tree and principal component analysis, we excluded the abnormal individuals and confirmed the parents and offspring as a whole for constructing the linkage map (Supplementary Figures). For female and male-specific linkage maps, 1427 and 1460 SNP markers were separately selected (Table 2).

**Table 2:** SNP markers for linkage mapping.

	Number
Detected SNP markers	9100
Filtered SNP markers	6674
SNP markers used for constructing female-specific linkage map	1427
SNP markers used for constructing male -specific linkage map	1460
SNP markers used for constructing the consensus linkage map	2508

**Figure 1:**
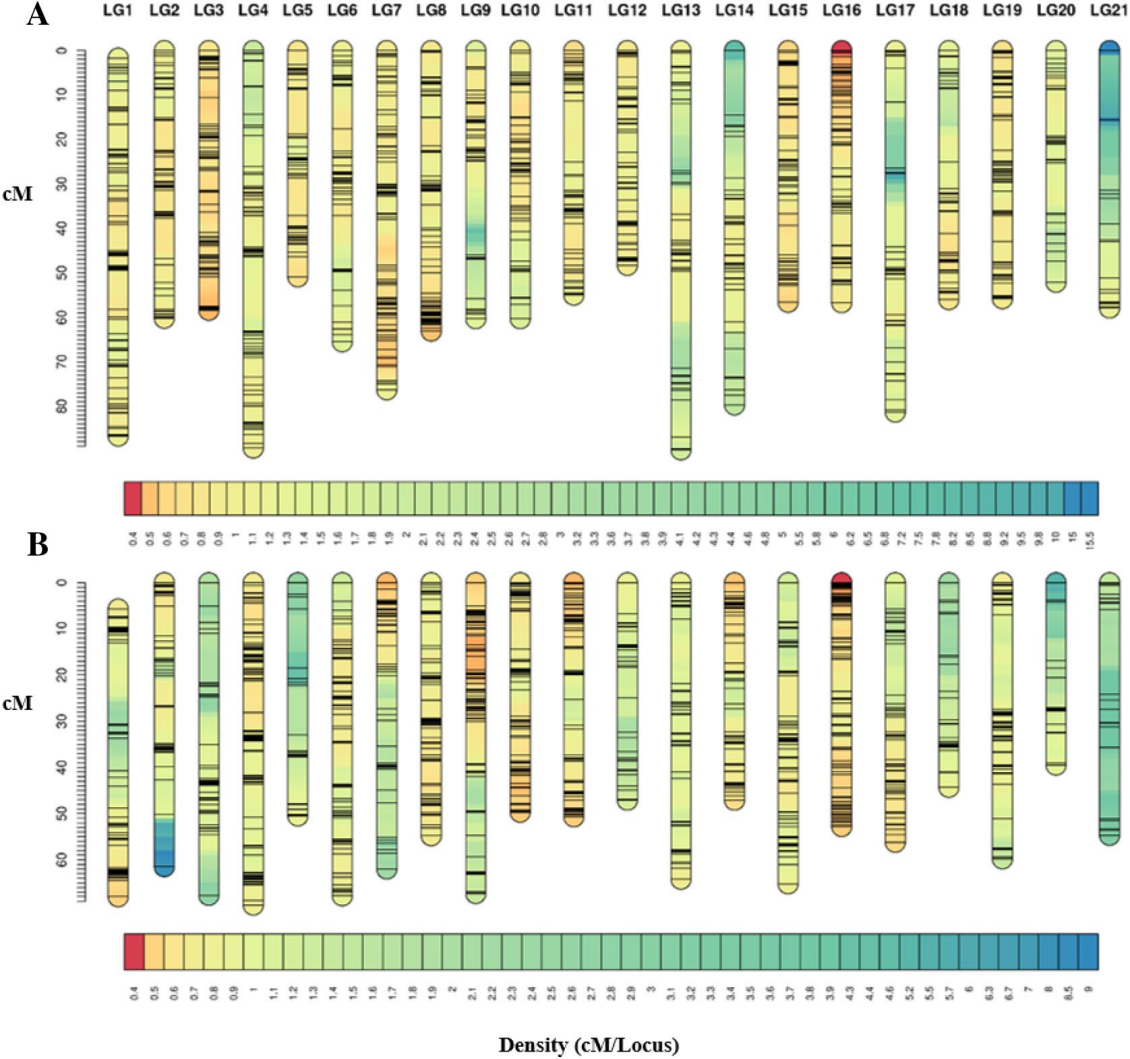
Genetic distance and marker distribution in two parental linkage maps. **(A)** The linkage map of the female parent, **(B)** the linkage map of the male parent. The scale plate on the left indicates genetic distance (cM as a unit). The below color module represents the distribution density of the SNP marker per cM.

The SNP markers were grouped to 21 LGs, corresponding to the chromosome number of *R. esculentu*^13^ (Fig. 1). The genetic lengths of the female and male-specific maps were 1360.29 and 1200.62 cM with an average marker interval of 0.95 cM and 0.82 cM, respectively (Fig. 1).

**Figure 2:**
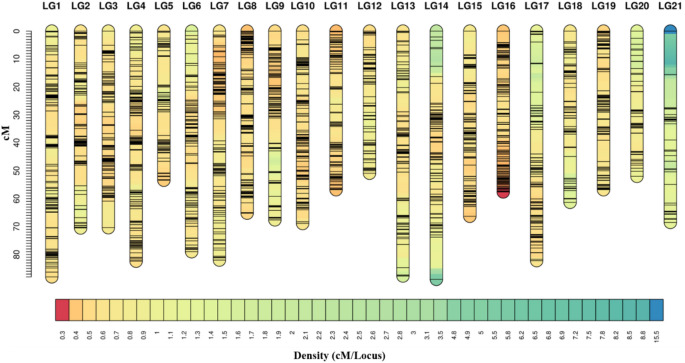
Genetic distance and marker distribution in the consensus linkage map of *R. esculentum*. The scale plate on the left indicates genetic distance (cM as a unit). The below color module represents the distribution density of the SNP marker per cM.

The consensus linkage map of *R. esculentum* was constructed using 2508 SNP markers with map coverage of 98.68% (Fig. 2; Supplementary Table S1). The genetic length of the consensus linkage map was 1456.34 cM with an average marker interval of 0.58 cM (Fig. 2; Supplementary Table S1). The longest LG is LG14 with a genetic length of 88.9 cM, 1.75-fold higher than that of the shortest LG20 (Fig. 2).

### QTL mapping

Six QTLs were detected for the growth of *R. esculentum*, including three for umbrella diameter and three for body weight (Table 3). These six QTLs located on four LGs, explaining 9.4 to 13.0% variation (Table 2). Two QTL regions for umbrella diameter that located on LG 14 and 15 are close to the QTL regions for body weight. In addition, the LOD value of the identified QTLs floated between 3.0 and 4.3 (Table 3).

**Table 3:** QTLs associated with umbrella diameter (UD) and body weight (BW) in *R. esculentum*.

Traits	Linkage group	Location (cM)	LOD value	Explained variation (%)
UD	13	42.40–41	3.1	9.6
UD	14	48.73	3.68	11.3
UD	15	40.4	4.30	13.0
BW	4	15.34	3.22	9.9
BW	14	49.45	3.4	10.5
BW	15	41.4	3.06	9.4

### Candidate genes related to growth traits

A total of 28 and 35 candidate genes in the QTL regions were identified associated with umbrella diameter and body weight, respectively (data not shown). Of these candidate genes, 27 genes were overlapped, associating with both umbrella diameter and body weight (Table 4). As we can see from Table 4, 22 genes have functional annotation and ten genes have metabolic roles, such as thermogenesis, mTOR signaling pathway, glutathione metabolism, biosynthesis of nucleotide sugars according to the KEGG database (Table 4). Due to the calcium-binding EGF-like (EGF_CA) domain, *RE13670* (sushi, von Willebrand factor type A, EGF and pentraxin domain-containing protein 1-like) was considered to play a key role in controlling the growth of *R. esculentum* (Table 4). Using online software NCBI-blast, we found RE13670 was most homologous to sushi, von Willebrand factor type A, EGF and pentraxin domain-containing protein 1-like of *Acropora digitifera* with a percent identity of 30.36%, supporting the accuracy of gene annotation. However, the metabolic roles of RE13670 were not found (Table 4).

**Table 4:** Candidate genes related to umbrella diameter and body weight of *R. esculentum.*

Gene name	Gene annotation	Conserved domain	KEGG pathway
*RE13677*	Myb-like protein X	SGNH_hydrolase	–
*RE13676*	CSC1-like protein	COG5594 super family	Thermogenesis
*RE13675*	–	–	–
*RE13674*	CREB-binding protein-like	zf-TAZ super family	TGF-beta signaling pathway; adherens junction; Wnt signaling pathway
*RE13673*	Gem-associated protein 8	GEMIN8 super family	–
*RE13672*	Transmembrane 9 superfamily member 3	Endomembrane protein 70	–
*RE13671*	Transcription factor IIIA	FOG	–
*RE13670*	sushi, von Willebrand factor type A, EGF and pentraxin domain-containing protein 1-like	Ephrin_rec_like; DUF5011 super family; IG_like; PLAT; FXa_inhibition; EGF_CA; Ephrin_rec_like super family	–
*RE13669*	-	CAP_ED	–
*RE13668*	eukaryotic translation initiation factor 5B	InfB super family	–
*RE03622*	Eukaryotic translation initiation factor 4 gamma 1-like, partial	PTZ00184; MIF4G; MA3 domain	–
*RE03621*	Nucleotide-binding oligomerization domain-containing protein 1-like	–	–
*RE03620*	Hypothetical protein CICLE_v10024023mg	MIF4G; CIDE-N; Med15 super family	–
*RE03619*	A-kinase anchor protein 7 isoform X3	AKAP7_NLS	–
*RE03618*	–	CIDE_N super familyat the N-terminus of the CIDE (cell death-inducing DFF45-like effector)	Apoptosis
*RE03617*	–	–	–
*RE03616*	Wnt4	Wnt super family	mTOR signaling pathway; Wnt signaling pathway
*RE03615*	Protein Wnt-4-like isoform X1	Wnt	mTOR signaling pathway; Wnt signaling pathway
*RE03614*	6-phosphogluconate dehydrogenase, decarboxylating-like	PRK09287 superfamily	Pentose phosphate pathway; glutathione metabolism; biosynthesis of secondary metabolites
*RE03613*	Serine/arginine-rich splicing factor 6-like isoform X1	RRM_SF super family	Spliceosome
*RE03612*	Hypothetical protein TRIADDRAFT_57615	RRM_SF super family; SF-CC1 super family	–
*RE03611*	Reduced wall acetylation 1-like	Cas1_AcylT super family	–
*RE03610*	Prohibitin-2-like	SPFH_prohibitin	–
*RE03609*	-	ICAT	–
*RE03608*	A disintegrin and metalloproteinase with thrombospondin motifs 9-like	Von Willebrand factor A	Focal adhesion; cell adhesion molecules; Rap1 signaling pathway; regulation of actin cytoskeleton
*RE03607*	Probable fructokinase-5	SIS super family	Biosynthesis of nucleotide sugars; lipopolysaccharide biosynthesis
*RE03606*	DNA fragmentation factor subunit beta-like	DFF40 super family; CIDE_N domain; cell death-inducing DFF45-like effector	Apoptosis

“–” indicated no information were found.

## Discussion

The molecular marker shows potential for investigating the growth of aquaculture animals in the genetic breeding industry^16^. SNP markers as one of the molecular markers were genotyped easily to construct the genetic linkage maps for guiding the genetic breeding of aquaculture animals^17^. Researchers have identified 1,034,708 SNPs in *R. esculentum* by transcriptome sequencing^15^, yet we only identified 9100 SNPs by 2b-RAD sequencing, much lower than that in the previous study^15^. The difference in SNP numbers between the two studies may be caused by the SNP analysis method. In the previous study, SNPs were detected by GATK2 software without reference genome while by RADtyping software according to *R. esculentum* genome in this study. Due to the simplicity and flexibility, the 2b-RAD method was extensively used for identifying SNPs and constructing high-density linkage maps for aquaculture animals^10,11,17–19^. By 2b-RAD method, Zhu et al. constructed the high-density linkage map of *Pseudobagrus ussuriensis* utilizing 7435 SNPs with a marker interval of 0.357 cM^17^. For *R. esculentum*, the marker interval of the linkage map is 0.58 cM at medium density, higher than that of *H. nobilis*^10^, *H. wyckioides*^11^ and *Channa argus*^19^, lower than that of *C. auratus*^8^, *P. ussuriensis*^17^, *Larimichthys crocea*^20^. The difference of marker interval between *R. esculentum* and the other aquaculture animals may attribute to the SNP numbers used for constructing linkage maps. For *P. ussuriensis*^17^ and *C. auratus*^8^, 7435 and 8487 SNPs were used for constructing the linkage map, which is 1.96-fold and 2.38-fold higher than that of *R. esculentum*, respectively*.* However, the identified SNPs in *R. esculentum* are very important markers for genetic breeding and this is the first report of linkage map in *R. esculentum*.

The high-density linkage map plays an important role in performing QTL mapping and finding genes related to the growth traits in aquatic animals^7,8,10,11^. Numerous QTLs about growth traits, such as body weight, body length, sex as well as disease resistance were identified based on the high-density linkage map^8,19–25^. For *Nibea albiflora*, 15 QTLs were detected associated with body weight, explaining 14.7–35.7% of the phenotypic variations^21^. For *C. auratus* at 2 months, eight QTLs in eight chromosomes were discovered associated with the body weight, explaining 10.1–13.2% of the phenotypic variations^8^. In this study, three QTLs distributed in three LGs were detected associated with body weight of *R. esculentum* and explained 9.4–10.5% of the phenotypic variations, following the result of *C. auratus*^8^. In addition, our studies showed that growth-related traits body weight and umbrella diameter in *R. esculentum* are positively correlated with the two close QTLs (Supplementary Table S2; Table 3).

Positional cloning of candidate genes with the help of QTL mapping may provide an efficient method for selective breeding in aquaculture animals^8,21,26^. We identified 27 candidate genes in LG 14 and 15 corresponding to the growth of *R. esculentum* and RE13670 showed the most possibility in controlling the growth due to the EGF_CA domain, following the growth-related genes reported in *C. auratus*^8^. For *C. auratus*, five candidate genes show potential for body weight and two genes (TGF-beta and EGF-like domain) may be the most promising according to their role in early growth and development of vertebrates^8^. EGF_CA domain is a calcium-binding EGF-like domain and needs calcium for performing biological function^27^. EGF_CA domain presented in extracellular (mostly animal) and membrane-bound^27^ and has three main roles, including protein–protein interactions, as a spacer unit and structural stabilization^28^. Although the functional significance of EGF_CA domain in aquaculture animals is unclear, it is worth confirming if EGF_CA domain is associated with the growth of *R. esculentum*. With the release of genomic data of *R. esculentum*^1,13^ and the development of biotechnology, the gene function studies of *R. esculentum* will be improved for studying genetic breeding.

## Conclusion

In this study, a total of 9100 SNP were identified and a high-density linkage map was constructed with a marker interval of 0.58 mM in of *R. esculentum* using the 2b-RAD method. Based on the linkage map, six QTLs were identified associated with the growth of *R. esculentum* and one candidate gene *RE13670* containing EGF_CA domain in LG14 may play the key role in controlling the growth of *R. esculentum*. Although one full-sib family of *R. esculentum* is limited*,* the identified SNPs and genes for growth will accelerate the MAS breeding of *R. esculentum*.

## Materials and methods

### Mapping family and DNA extraction

A full-sib family of *R. esculentum* was constructed in Yingkou City, Liaoning province, China. We selected the two parents with a large difference in growth from the breeding pond and cultured their offspring in the nursery pond. The two parents and random 150 offspring at seven-month-old (juvenile jellyfish) were chosen for sequencing. The body weight and umbrella diameter of the offspring were measured (Supplementary Table S1). The genomic DNA was extracted referred to the previous method^13^. The DNA quality was measured by Qingdao OE Biotech Co., Ltd.

### 2b-RAD sequencing

The 2b-RAD libraries of *R. esculentum* specimens were constructed at Qingdao OE Biotech Co., Ltd., following the published method^29^. Firstly, 100–200 ng genomic DNA was digested by 1 U BsaXI (New England Biolabs). Secondly, the ligation reaction was conducted to add specific adaptors to the digested genomic DNA. Thirdly, the ligation products were amplified in MyCycler thermal cyclers (Bio-Rad). Fourthly, the PCR products were purified using a MinElute PCR Purification Kit and digested using SapI (New England Biolabs). Fifthly, the digested products were transferred to the tube containing magnetic beads for incubation and then transferred the supernatant to a new tube for ligation using T4 DNA ligase (New England Biolabs). After that, the ligation products were purified and barcodes were introduced by PCR using barcode-bearing primers. Finally, PCR products were purified and pooled for sequencing using the Illumina Novaseq 6000 PE150 sequencing platform.

The raw data of 2b-RAD sequencing were trimmed for getting the high-quality data, and then the high-quality data were aligned to reads. The reads with the BsaXI site were extracted and aligned to the reference reads using SOAP (version 2.21)^30^. The reference reads were extracted from *R. esculentum* genome (NCBI Genome ID: 56778) after electronic digestion using the BsaXI enzyme.

### SNP detection and filter

The aligned data were used to detect SNP by RADtyping software^31^. The maximum-likelihood (ML) algorithm was used to detect homozygote or heterozygote in co-dominant markers. SNPs were filtered with the following criteria: (1) Segregating markers that could be genotyped over 80% of the progenies were retained; (2) Markers with a minor allele frequency (MAF) less than 0.01 were discarded; (3) Polymorphic loci that contain more than two alleles were excluded; (4) The aligned reads with more than two SNPs were discarded. Based on the detected SNPs, we constructed neighbor-joining tree using treebest (version 4.1)^32^ and performed principal component analysis using ADMIXTURE (version 1.3.0)^33^ for confirming the species as a full-sibling family. SNP markers were annotated using the software SnpEff (version 4.1)^34^.

### Linkage map construction

After filtering, the linkage group was divided with the LOD value of 2–15. Marker distances were calculated using Kosambi’s mapping function^35^. The male and female-specific linkage maps were constructed by software JoinMap (v5.0)^36^. The male-specific linkage map was constructed using paternal heterozygous genotype and maternal heterozygous and homozygous genotype. The female-specific linkage map was constructed using maternal heterozygous genotype and paternal heterozygous and homozygous genotype. The consensus genetic linkage map was constructed by merging male and female-specific linkage maps using the software MergeMap (http://138.23.178.42/mgmap/) ^37^.

### QTL mapping of growth traits

Based on the consensus linkage map, QTL mapping analyses of body weight and umbrella diameter in *R. esculentum* were performed by software MapQTL (v6.0)^38^. The interval mapping method was used for genome-wide QTL analysis and every one cM on each LG was scanned for searching the possible QTL. LOD threshold value at 95% level was calculated via 1000 permutation tests for each trait and QTL. LOD score of QTL that was greater than the LOD threshold value (2.5) at 95% level was declared significant.

### Candidate genes associated with growth

The genes located on the up-and down-stream 500 Kb distance of the associated genomic region of body weight and umbrella diameter were detected^13,39^. We ascertained the candidate genes by combining their function annotation according to NR and KEGG database^40^ and analyzed results via online software NCBI-blast (https://blast.ncbi.nlm.nih.gov/Blast.cgi) and conserved domain search service (https://www.ncbi.nlm.nih.gov/Structure/cdd/wrpsb.cgi).

## Supplementary Information

Supplementary Information.

Supplementary Figures.

Supplementary Tables.

## Data Availability

The raw sequencing data were available at the National Genomics Data Center under Accession Number PRJCA007242. The other data are available from the corresponding author upon reasonable request.
