# Supplementary material for: Linkage mapping and QTL analysis of growth traits in Rhopilema esculentum
Source: Sci Rep. 2022 Jan 10;12:471. doi: 10.1038/s41598-021-04431-0 (PMC8748825; doi:10.1038/s41598-021-04431-0)
Supplement: Supplementary file 1 — Supplementary Information. [file 41598_2021_4431_MOESM1_ESM.docx]

> RE13670

MFSLCIAILVLSFLGSSDGKTYQLKVRTSKHIGAGTGENVKITLFGSNGKKVFKEYNGWGLHMFDFGMTNELELEGEDIGSIIDIQAQIVKTGRYLDKWRLFWIKVFADGDAFKGTFEYTFRSDKETSNAKLFEILGCREGYEPTEIGGRKTCADRNECLVTCKGPGQECVNIPGSYRCNCKPGFYFDGSECRDYDECLQHDQDPCSYPNAHCKNLPGSYECRCNRGYEGDYHSCQDKDECKLNLHSCSQKCVNIVGGYRCDCNTGFKLAVDGKTCLDADECKGAHSCNLTTSVCYDEYGSHRCICKAGYKEDTTDPKRKTCIPIECQPLSNMEKSIILSPPRCMNGKNIYGDECSVKCAPGYELDAGSAKTLRCTSFGFVNINGNMPMCKPKPCGKLAVPNFGFTVPPSCSSVGATQGSQCYIFCDHGFVLAGERAYTCDQQKYDKDPSKTMCVRIPKIECPSDVTVALPQDSSRVALDNNFPYFRTNVRRDQISSNIAGIGPSYEFPLGHNVVVYKATNEVNQTDKCSFVVSVEDRSPPVLEFCPSSFTVVSKGAVPVNVTWEEPRFKDNVGVQSVIASSKSGELRSPTDFTVLYRAFDASRNMATCKFTVSFKVLRCDFDSIPGGDQLIQKSCMNMGTSHLCMAQCVPTKTFNVINSKHMFLKFMWSCVKADFEVDQMPDCVDFQPKNGKPCPAGSTDVNDYRSGLPVSKCARGTYYVNGSCTDCPTSMYQPLEGSLSCEKCKSGYGTSTKRNKLSTDCKEQCPLGYFSTDGLSDGQSSCEMCPKNTYADKFGSKSCLRCPNGTTTDGSGRTSISACRYAPKNIRMVPGGTIEVSAGEKVHFDCFADGNPMPFVHIRKQQSQANPMTHSLQPVAAPNSNTKGIRYMISSASIHDSDYYVCRAENNKGIIYNKIQLVVTEGSGMSSIG
