# Supplementary material for: Linkage mapping and QTL analysis of growth traits in Rhopilema esculentum
Source: Sci Rep. 2022 Jan 10;12:471. doi: 10.1038/s41598-021-04431-0 (PMC8748825; doi:10.1038/s41598-021-04431-0)
Supplement: Supplementary file 2 — Supplementary Figures. [file 41598_2021_4431_MOESM2_ESM.docx]

Title: Linkage mapping and QTL analysis of growth traits in *Rhopilema esculentum*

Authors: Bailing Chen^1^, Yulong Li^1^, Meilin Tian^1^, Hao Su^1^, Wei Sun^1^& Yunfeng Li^1*^

Affiliations: ^1^Liaoning Ocean and Fisheries Science Research Institute, 50 Heishijiao St., Dalian, Liaoning 116023, China

* Corresponding authors: Yunfeng Li^1*^ yunfengli@126.com

**Figure legends**

Figure S1. Phylogenetic tree of *Rhopilema esculentum* species

Figure S2. Principal component analysis of SNP data from *Rhopilema esculentum* species


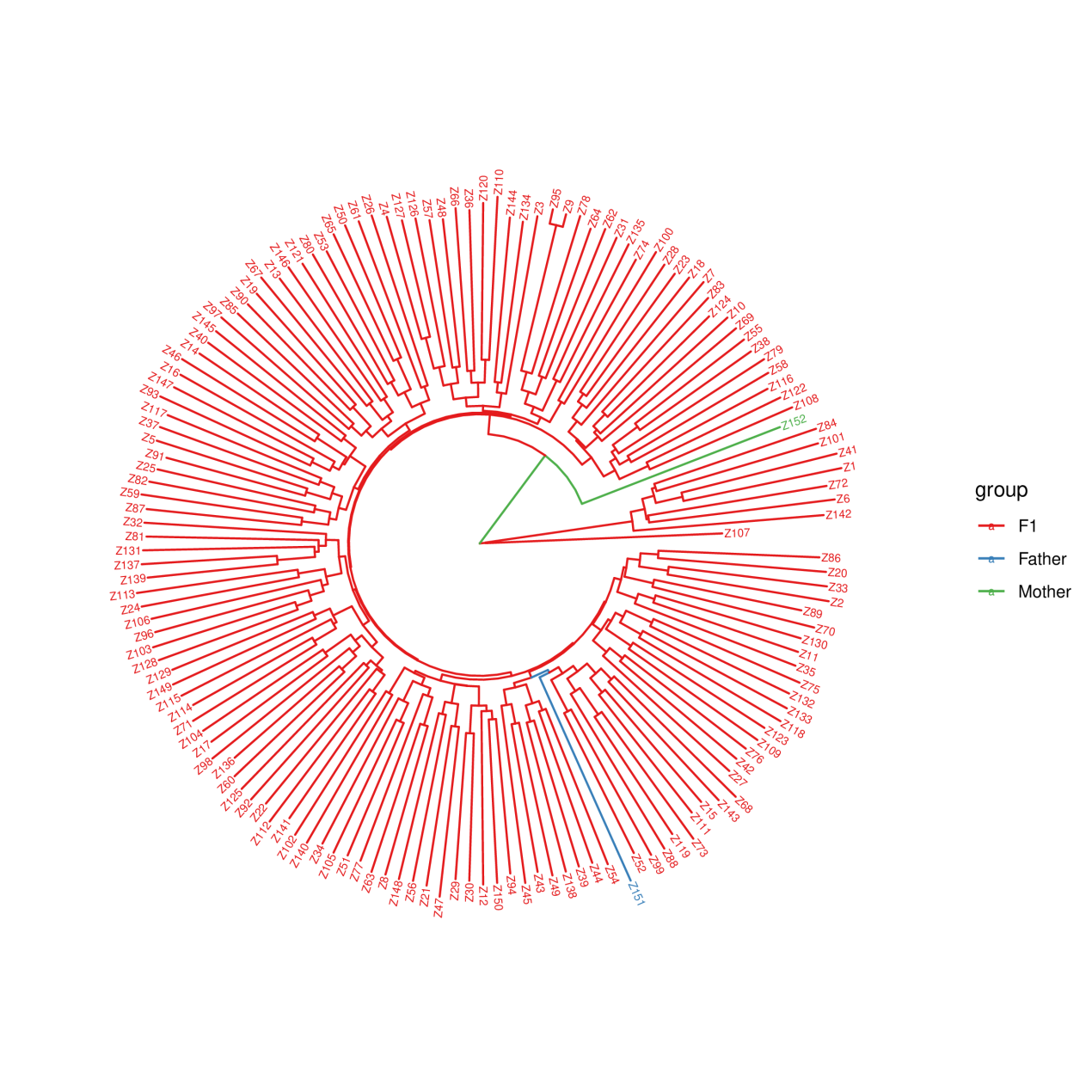


**Figure S1**


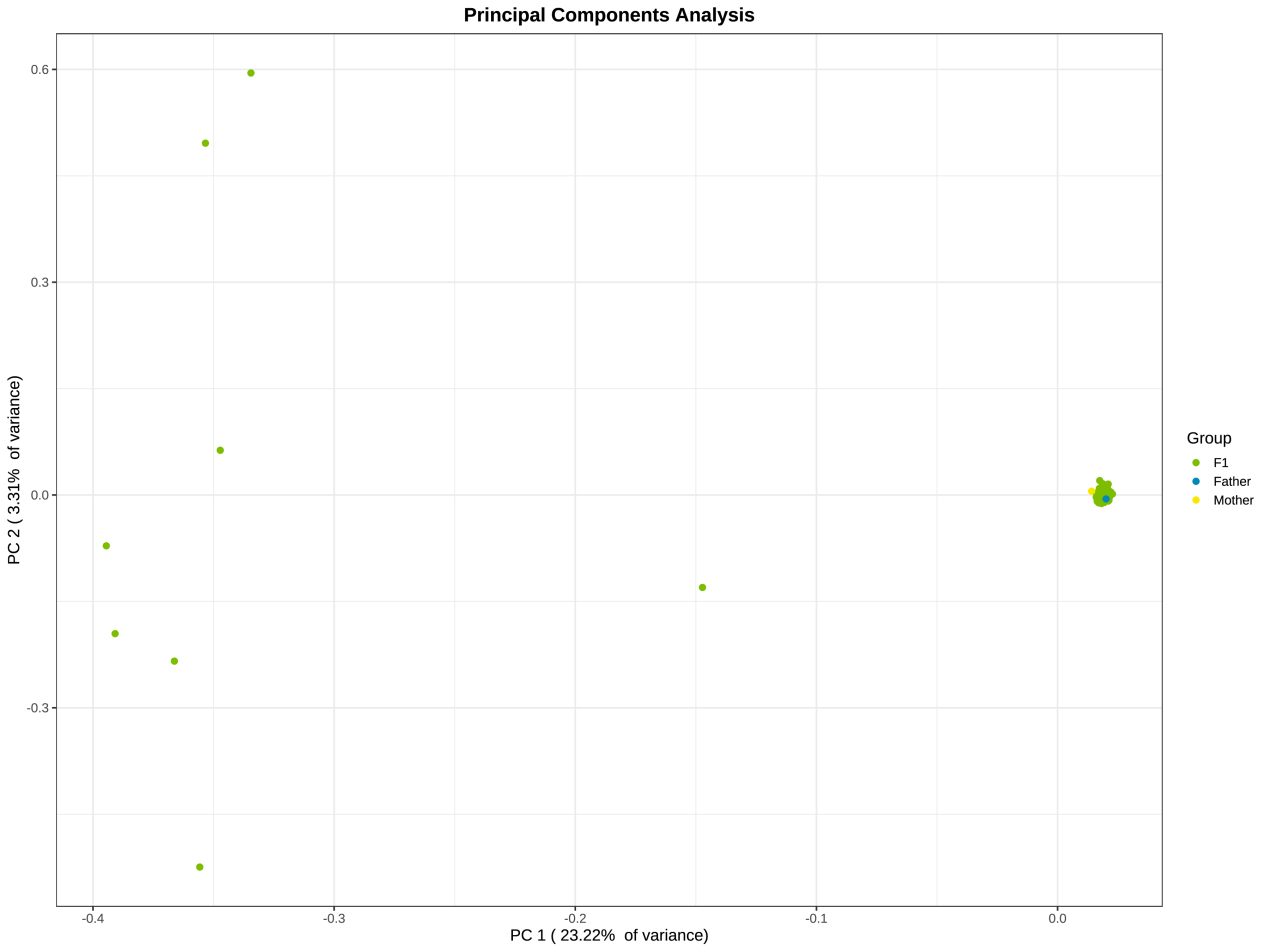


**Figure S2**
