# Supplementary material for: Linkage mapping and QTL analysis of growth traits in Rhopilema esculentum
Source: Sci Rep. 2022 Jan 10;12:471. doi: 10.1038/s41598-021-04431-0 (PMC8748825; doi:10.1038/s41598-021-04431-0)
Supplement: Supplementary file 3 — Supplementary Tables. [file 41598_2021_4431_MOESM3_ESM.docx]

Title: Linkage mapping and QTL analysis of growth traits in *Rhopilema esculentum*

Authors: Bailing Chen^1^, Yulong Li^1^, Meilin Tian^1^, Hao Su^1^, Wei Sun^1^& Yunfeng Li^1*^

Affiliations: ^1^Liaoning Ocean and Fisheries Science Research Institute, 50 Heishijiao St., Dalian, Liaoning 116023, China

* Corresponding authors: Yunfeng Li^1*^ yunfengli@126.com

Table S1 Summary of the consensus genetic linkage map in *R. esculentum*

| Linkage group | Mapped markers | Distinct positions | Genetic length (cM) | Marker interval (cM) |
| --- | --- | --- | --- | --- |
| LG1 | 150 | 140 | 88.02 | 0.59 |
| LG2 | 130 | 118 | 70.54 | 0.54 |
| LG3 | 137 | 127 | 70.37 | 0.51 |
| LG4 | 148 | 142 | 82.38 | 0.56 |
| LG5 | 94 | 86 | 53.43 | 0.57 |
| LG6 | 129 | 119 | 78.95 | 0.61 |
| LG7 | 158 | 144 | 81.98 | 0.52 |
| LG8 | 146 | 138 | 65.23 | 0.45 |
| LG9 | 122 | 113 | 67.6 | 0.55 |
| LG10 | 140 | 130 | 68.86 | 0.49 |
| LG11 | 128 | 122 | 56.78 | 0.44 |
| LG12 | 75 | 70 | 50.89 | 0.68 |
| LG13 | 131 | 120 | 87.66 | 0.67 |
| LG14 | 120 | 105 | 88.9 | 0.74 |
| LG15 | 130 | 121 | 66.32 | 0.51 |
| LG16 | 159 | 149 | 57.61 | 0.36 |
| LG17 | 115 | 109 | 82.17 | 0.71 |
| LG18 | 83 | 76 | 61.3 | 0.74 |
| LG19 | 114 | 109 | 56.78 | 0.5 |
| LG20 | 58 | 56 | 52.04 | 0.9 |
| LG21 | 41 | 37 | 68.53 | 1.67 |
| Total | 2508 | 2331 | 1456.34 | 0.58 |
| Expected genetic length | |  | 1475.89 |  |
| Coverage |  |  | 98.68% |  |

Table S2 Statistical analysis of body weight and umbrella diameter in F1 offspring of *R. esculentum*

| Phenotype | Min | Max | Mean | STDEV | C.V (%) |
| --- | --- | --- | --- | --- | --- |
| Body weight | 2.70 | 33.50 | 9.14 | 4.55 | 49.79 |
| Umbrella diameter | 3.20 | 7.00 | 4.47 | 0.75 | 16.82 |
